# Engineered Nanomaterials in the Environment

**DOI:** 10.3390/nano6060106

**Published:** 2016-06-06

**Authors:** Jonathan D. Judy, Paul Bertsch

**Affiliations:** 1 Commonwealth Science and Industry Research Organization (CSIRO) Land and Water, Waite Campus, Urrbrae 5064, Australia; 2 CSIRO Land and Water, Ecosciences Precinct, 41 Boggo Road, Dutton Park 4102, Australia; paul.bertsch@csiro.au

This Special Issue of *Nanomaterials*, “Engineered Nanomaterials in the Environment”, is comprised of one communication and five research articles. Engineered nanomaterials (ENMs) composed of materials such as ZnO, TiO_2_, Ag and CuO are being incorporated into consumer products including sunscreens, cosmetics, pharmaceuticals and textiles. The ENMs in these consumer products will be released into waste streams during the products’ life cycles and will concentrate in sewage sludge during wastewater treatment. In areas where biosolids are used as fertilizer, land application of biosolids is a pathway by which ENMs will enter agroecosystems. Furthermore, ENMs are also increasingly being investigated for use in the development of novel pesticides, nutrient delivery systems and fertilizers [1]. These agricultural applications present the possibility of widespread, large volume discharge of as-manufactured ENMs into agroecosystems. As questions remain over the potential environmental impact of ENMs, scientific and community concerns persist over their human and environmental safety.

ENMs from consumer products discharged into wastewater will be transformed into aged ENMs prior to being introduced into the environment [2]. The usefulness of data from experiments using pristine ENMs to characterize the risk associated with the discharge of ENMs via this pathway is questionable. Recognizing this, Mallevre *et al.* examined the toxicity of pristine and aged Ag ENMs to the bioluminescent bacteria *Pseudomonas putida* [3]. Bacteria were exposed in crude and final wastewater collected from four wastewater treatment plants in Scotland. Aging of Ag ENMs reduced toxicity, a result consistent with other recent work [4,5,6], with the IC_50_ at 8 weeks of aging being almost twice that observed for un-aged ENMs in final wastewater and approximately three times that observed for un-aged ENMs in crude wastewater.

As previously mentioned, ENMs are increasingly being evaluated to determine whether they can be used to develop more effective fertilizers or pesticides. Wagner *et al.* reported the results of one such study examining the effectiveness of Zn and ZnO ENMs to treat and prevent infection by the tobacco pathogen, *Peronospora tabacina* [7]. Both types of ENMs inhibited leaf infection and *P. tabacina* spore germination to a degree that could not be explained by the presence of dissolved Zn. Also in this Special Issue, Wang *et al.* reports on the use of organic phase change ENMs to label agrochemicals and prevent product counterfeiting [8]. Counterfeiting of agrochemicals poses a serious risk to agroecosystems, as counterfeited products are often made with poor quality reactants and may contain unknown or banned constituents [8]. Once introduced into agroecosystems, the environmental risk of these unknown and banned chemicals is unknown. Wang *et al.* describes how polymer-encapsulated ENMs composed of paraffin wax and fatty acids can be synthesized and used as a highly covert, easily detectable and widely applicable product label when mixed with a variety of agrochemicals.

The bioavailability and ultimate fate of ENMs discharged into the environment through either the biosolids or agricultural pathway remains unclear. Much of the research to date concerning the bioavailability of ENMs has been conducted in relatively simple systems and the potential exists that variables that may fundamentally alter ENM bioavailability in environmentally realistic exposure settings are not being adequately considered. One variable hypothesized to be potentially influential is plant root colonization by arbuscular mycorrhizal fungi (AMF), which could present a pathway by which ENMs could be trafficked into plants. In this Special Issue, Judy *et al.* exposed two varieties of tomato, a mutant that does not allow AMF colonization and its progenitor, to Au ENMs in soil. Spatial analysis of root cross-sections revealed Au accumulated at the root rhizoplane. However, bulk analysis of the plant tissues indicated that colonization by AMF did not increase the bioavailability of ENMs from soil [9].

The degree to which ENMs will resist aggregation in the environment will be highly influential to both their mobility as well as their bioavailability. Mui *et al.* investigated the colloidal stability of Al_2_O_3_ ENMs as a function of different environmental variables, finding that pH, ionic strength and the presence of humic acid and inorganic colloids strongly influenced ENM aggregation [10]. Also reported was the observation that when pH < point of zero charge (PZC), basal planes of clay particles were the dominant sites of interaction with ENMs. However, when pH was approximately equal to the PZC, edge sites were the dominant interaction sites. 

These research studies add information to the growing body of work regarding the environmental safety of ENMs, which is being used by regulatory bodies around the world to assess the risk posed to the environment by ENMs. The Biocidal Product Regulation (BPR) is a regulation in the European Union that contains provisions for biocides containing ENMs. This regulation and challenges associated with its implementation are discussed in this Special Issue by Brinch *et al.* [11]. This article discusses how the lack of standard methods for evaluating ENM risk presents a major challenge for regulators and industry and suggests that standard practices regarding ENM exposure suspension preparation and ecotoxicological tests be adopted.

In this Special Issue, diverse topics covering several different aspects dealing with the safety of nanotechnology, including ecotoxicology [3], bioaccumulation pathways [9], environmental behavior [10], potential applications [7,8] and regulation [11], are discussed. These articles simultaneously illustrate the enormous promise, potential risk and regulatory implications of the use of ENM-based technologies and demonstrate the need for additional research to ensure that the transformational potential of nanotechnology is responsibly fulfilled.
